# Oxygen‐Evolving Mesoporous Organosilica Coated Prussian Blue Nanoplatform for Highly Efficient Photodynamic Therapy of Tumors

**DOI:** 10.1002/advs.201700847

**Published:** 2018-02-22

**Authors:** Zhen Lu Yang, Wei Tian, Qing Wang, Ying Zhao, Yun Lei Zhang, Ying Tian, Yu Xia Tang, Shou Ju Wang, Ying Liu, Qian Qian Ni, Guang Ming Lu, Zhao Gang Teng, Long Jiang Zhang

**Affiliations:** ^1^ Department of Medical Imaging Jinling Hospital School of Medicine Nanjing University Nanjing 210002 Jiangsu P. R. China; ^2^ Department of Interventional Radiology First Affiliated Hospital of Nanjing Medical University Nanjing 210029 Jiangsu P. R. China; ^3^ Department of Urology Tongji Hospital Tongji Medical College Huazhong University of Science and Technology Wuhan 430030 Hubei P. R. China; ^4^ State Key Laboratory of Analytical Chemistry for Life Science School of Chemistry and Chemical Engineering Nanjing University 210093 Nanjing P. R. China

**Keywords:** chlorin e6, oxygen‐evolving nanoplatforms, periodic mesoporous organosilica, photodynamic therapy, prussian blue

## Abstract

Oxygen (O_2_) plays a critical role during photodynamic therapy (PDT), however, hypoxia is quite common in most solid tumors, which limits the PDT efficacy and promotes the tumor aggression. Here, a safe and multifunctional oxygen‐evolving nanoplatform is costructured to overcome this problem. It is composed of a prussian blue (PB) core and chlorin e6 (Ce6) anchored periodic mesoporous organosilica (PMO) shell (denoted as PB@PMO‐Ce6). In the highly integrated nanoplatform, the PB with catalase‐like activity can catalyze hydrogen peroxide to generate O_2_, and the Ce6 transform the O_2_ to generate more reactive oxygen species (ROS) upon laser irradiation for PDT. This PB@PMO‐Ce6 nanoplatform presents well‐defined core–shell structure, uniform diameter (105 ± 12 nm), and high biocompatibility. This study confirms that the PB@PMO‐Ce6 nanoplatform can generate more ROS to enhance PDT than free Ce6 in cellular level (*p* < 0.001). In vivo, the singlet oxygen sensor green staining, tumor volume of tumor‐bearing mice, and histopathological analysis demonstrate that this oxygen‐evolving nanoplatform can elevate singlet oxygen to effectively inhibit tumor growth without obvious damage to major organs. The preliminary results from this study indicate the potential of biocompatible PB@PMO‐Ce6 nanoplatform to elevate O_2_ and ROS for improving PDT efficacy.

## 1 Introduction

Photodynamic therapy (PDT) has been proven to be a potential therapeutic strategy for cancers^1^ via the reaction between photosensitizers and oxygen (O_2_) under laser to generate cytotoxic reactive oxygen species (ROS) for killing cancer cells.^2^, ^3^, ^4^, ^5^, ^6^ Compared to traditional cancer therapy strategies, such as surgery, chemotherapy, and radiotherapy, PDT emerges as a promising treatment method with less invasiveness, fewer side effects, and higher selectivity and efficacy.^2^, ^4^, ^7^, ^8^, ^9^ Because the PDT process is dependent on O_2_ concentration, tumor hypoxia originating from the rapid tumor growth and abnormal tumor vessels^3^, ^10^, ^11^ limits the efficacy of PDT and promotes therapeutic resistance and cancer progression.^12^, ^13^

Many efforts have been made to supply oxygen for ensuring PDT efficacy. Red blood cells have been used as natural O_2_ carriers for PDT in hypoxic cancers.^14^ However, the spontaneous exchange of O_2_ and carbon dioxide (CO_2_) results insufficient O_2_ delivery.^15^ Hyperbaric oxygen therapy, as another method to relieve cancer hypoxia, is limited by its intrinsic side effects, such as hyperoxic seizures and barotraumas.^2^, ^3^ Recently, nanomaterials have been synthesized to deliver or generate O_2_ molecules, including perfluorocarbon, CaO_2_, MnO_2_, and hydrogen peroxide (H_2_O_2_) or catalase loaded nanoparticles.^2^, ^3^, ^15^, ^16^, ^17^, ^18^, ^19^, ^20^ Although those strategies are beneficial to PDT by ameliorating hypoxia in some degree, several disadvantages exist, such as poor biocompatibility, the need for exogenous activation, complex synthesis procedures, cytotoxicity of concentrated H_2_O_2_, and short half‐life of catalase.^2^, ^15^ Therefore, it is necessary to design simple and biocompatible nanoplatforms that evolve oxygen continuously without exogenous activation and then generate more ROS under laser for PDT.

Prussian blue (PB) nanoparticles are with excellent biocompatibility, which have been proved by U.S. Food and Drug Administration (FDA) for clinical application.^21^, ^22^, ^23^ In addition to the ability for photothermal therapy, magnetic resonance (MR), and photoacoustic (PA) imaging,^21^, ^22^ PB has been proven with catalase‐like activity to catalyze hydrogen peroxide into oxygen.^24^ It is reported that hydrogen peroxide is abundant in cancer microenvironment (with concentrations ranging from 100 × 10^−6^ m to 1 × 10^−3^ m) and is an appropriate source to produce O_2_ within tumors.^2^, ^25^, ^26^ However, the catalase‐like activity of PB nanoparticles has not been used for enhancing PDT.

Periodic mesoporous organosilica (PMO) is a class of promising mesoporous materials with organic groups directly incorporated into mesoporous frameworks, which holds high stability, biocompatibility, and biodegradability.^27^, ^28^ In this work, we chose PMO to coat the prussian blue core for loading photosensitizers. The 1,4‐bis(triethoxysily) propane tetrasulfide [TESPTS, (RO)_3_Si—CH_2_CH_2_CH_2_—S—S—S—S—CH_2_CH_2_CH_2_—Si(OR)_3_] was used as organosilica resource to coat PB nanoparticles, which made the PMO shells containing disulfide bonds in the framework.^29^ The acquired disulfide bonds can be reduced to thiol groups for subsequently linking PDT agents for tumor treatment.

In this work, we load a photosensitizer, chlorin e6 (Ce6), into periodic mesoporous organosilica coated prussian blue nanoparticles (PB@PMO) to develop an integrated, simple, and biocompatible platform for supplying O_2_ and generating singlet oxygen (^1^O_2_) for photodynamic therapy. The periodic mesoporous organosilica shell facilitates loading and delivering photosensitizers and the PB core can effectively catalyze H_2_O_2_ into O_2_ which sequentially receives the energy transferred by Ce6 from lasers to generate singlet oxygen for PDT. Owing to the PB core, this nanoparticle can also act as an MR and PA imaging contrast agent. We confirm the ability of the PB@PMO‐Ce6 in decomposing H_2_O_2_ into O_2_. As expected, this nanosystem can obviously elevate ROS for efficient PDT both in vitro and in vivo. The mice tumor volume, terminal‐deoxynucleoitidyl transferase mediated nick end labeling (TUNEL) and hematoxylin and eeosin (H&E) staining analysis indicate that the PB@PMO‐Ce6 nanoplatform can effectively inhibit and destroy tumor with satisfied biocompatibility. To the best of our knowledge, it is the first time to make use of the catalase‐like activity of PB‐based nanomaterials to enhance photodynamic therapy.

## 2 Results and Discussion

### 2.1 Characterization

**Figure 1:**
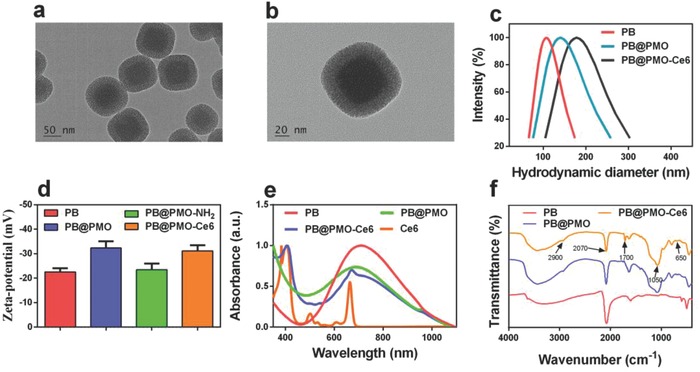
Characterization of PB@PMO‐Ce6 nanoplatforms. a,b) TEM images of PB@PMO; c) hydrodynamic diameters of PB, PB@PMO, and PB@PMO‐Ce6; d) zeta potentials of PB, PB@PMO, PB@PMO‐NH_2_, and PB@PMO‐Ce6; e) UV–vis spectra of PB, PB@PMO, and PB@PMO‐Ce6 and Ce6; f) FT‐IR spectra of PB, PB@PMO, and PB@PMO‐Ce6.

The transmission electron microscopy (TEM) image shows that the PB@PMO presents a well‐defined core–shell structure, demonstrating PB has been successfully coated with PMO (**Figure** **1**a,b). The diameter of the PB@PMO is 105 ± 12 nm with relatively uniform size distribution. The hydrodynamic diameters of PB, PB@PMO, and PB@PMO‐Ce6 are about 100, 133, and 170 nm, respectively (Figure 1c). Zeta potential of PB is −22.6 ± 1.5 mV and becomes −32.4 ± 2.6 mV after coated with PMO shells, which is in accordance with previous reported PMO zeta potential.^21^ After conjugating amino and Ce6, the zeta potential of the PB@PMO changes to −23.4 ± 2.6 mV and −31.1 ± 2.3 mV, respectively, which is attributed to the positive charge of amino and the negative charge of Ce6 (Figure 1d). The UV–vis absorbance spectrum of the PB@PMO‐Ce6 shows typical absorption peaks at 404 and 660 nm (Figure 1e), validating the modification of Ce6. The absorption peak at about 710 nm is due to the coated PB core. The loading capacity of the PB@PMO for Ce6 is measured to be 60 µg mg^−1^ (1 mg of PB@PMO loads 60 µg of Ce6) by using Ce6 UV calibration curve at 404 nm (Figure S1, Supporting Information). The Fourier transform infrared (FT‐IR) spectra of the PB, PB@PMO, and PB@PMO‐Ce6 display a vibration peak at 2070 cm^−1^, which is assigned the CN groups of PB. The PB@PMO and PB@PMO‐Ce6 also show the Si—O—Si vibration peak at 1050 cm^−1^, C—S band at 650 cm^−1^, and C—H band at 2900 cm^−1^,^21^, ^30^ confirming that the PB nanoparticles are successfully coated with PMO shells. The C=O vibration peak at 1700 cm ^−1^ in PB@PMO‐Ce6 further indicates successful Ce6 conjugation (Figure 1f).^31^, ^32^, ^33^ Nitrogen adsorption–desorption isotherms of the PB@PMO presented a typical IV curve, suggesting the mesoporous structure of the PMO shells (Figure S2a, Supporting Information). The surface area, pore volume, and pore size were 1337 m^2^ g^−1^, 0.65 cm^3^ g^−1^, and 2.3 nm, respectively (Figure S2b, Supporting Information).

### 2.2 Evaluating Catalase‐Like Activity of PB@PMO‐Ce6

**Figure 2:**
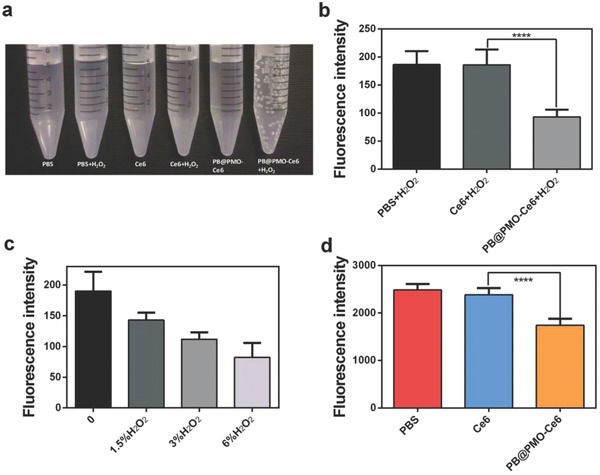
Evaluating catalase‐like activities of PB@PMO‐Ce6. a) The generation of oxygen gas bubbles in different groups; b) average fluorescence intensity of [Ru(dpp)_3_]Cl_2_‐added mixture containing H_2_O_2_ and PBS, Ce6 or PB@PMO‐Ce6, respectively; c) average fluorescence intensity of [Ru(dpp)_3_]Cl_2_‐added PB@PMO‐Ce6 solution incubated with H_2_O_2_ at different concentrations; d) average intracellular fluorescence intensity of [Ru(dpp)_3_]Cl_2_‐loaded U87MG cells incubated with PBS, Ce6, or PB@PMO‐Ce6, respectively.

The catalase‐like activity of the PB@PMO‐Ce6 was evaluated in an H_2_O_2_ solution. After the well‐mixed solution of PB@PMO‐Ce6 and diluted H_2_O_2_ is incubated at 37 °C for 30 min, obvious bubbles are observed, while negligible bubbles are found in other groups (**Figure** **2**a). Compared to Ce6 only, the O_2_ level in PB@PMO‐Ce6 group increases significantly (*p* < 0.0001), presenting as the reduced fluorescence intensity of the O_2_ probe ([Ru(dpp)_3_]Cl_2_) (Figure 2b). Further, the O_2_ amount elevates gradually along with the concentration of H_2_O_2_ when incubated with PB@PMO‐Ce6 (Figure 2c). Next, the detection of O_2_ amount demonstrates that the catalase‐like activity of the PB@PMO‐Ce6 is also effective in U87MG cells (Figure 2d) (*p* < 0.0001).

### 2.3 Detecting Reactive Oxygen Species In Vitro

**Figure 3:**
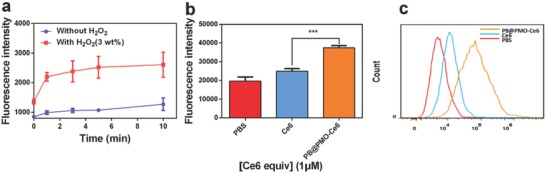
Detecting the generation of ROS. a) ^1^O_2_ production of PB@PMO‐Ce6 (1 × 10^−6^ m Ce6 equiv.) with or without H_2_O_2_ (3 wt%) under different laser irradiation time periods; b,c) the generation of ROS in U87MG cells incubated with different agents and then received laser irradiation (660 nm, 1 W cm^−2^, 5 min) measured by a microplate reader and a flow cytometry, respectively.

ROS is the most critical effector in PDT, so the ^1^O_2_ levels in the presence of PB@PMO‐Ce6 with or without diluted H_2_O_2_ were measured after irradiation by a 660 nm laser for different time (**Figure** **3**a). The fluorescence intensity elevates gradually along with time. Obviously, the generation rate and total amount of ^1^O_2_ are higher in the presence of PB@PMO‐Ce6 and H_2_O_2_ than PB@PMO‐Ce6 only (Figure 3a). On cell level, the fluorescence intensity is higher when cells are incubated with PB@PMO‐Ce6 than Ce6 (*p* < 0.001), which further demonstrates the ability of PB@PMO‐Ce6 to generate more ROS (Figure 3b,c).

### 2.4 Cytotoxicity

**Figure 4:**
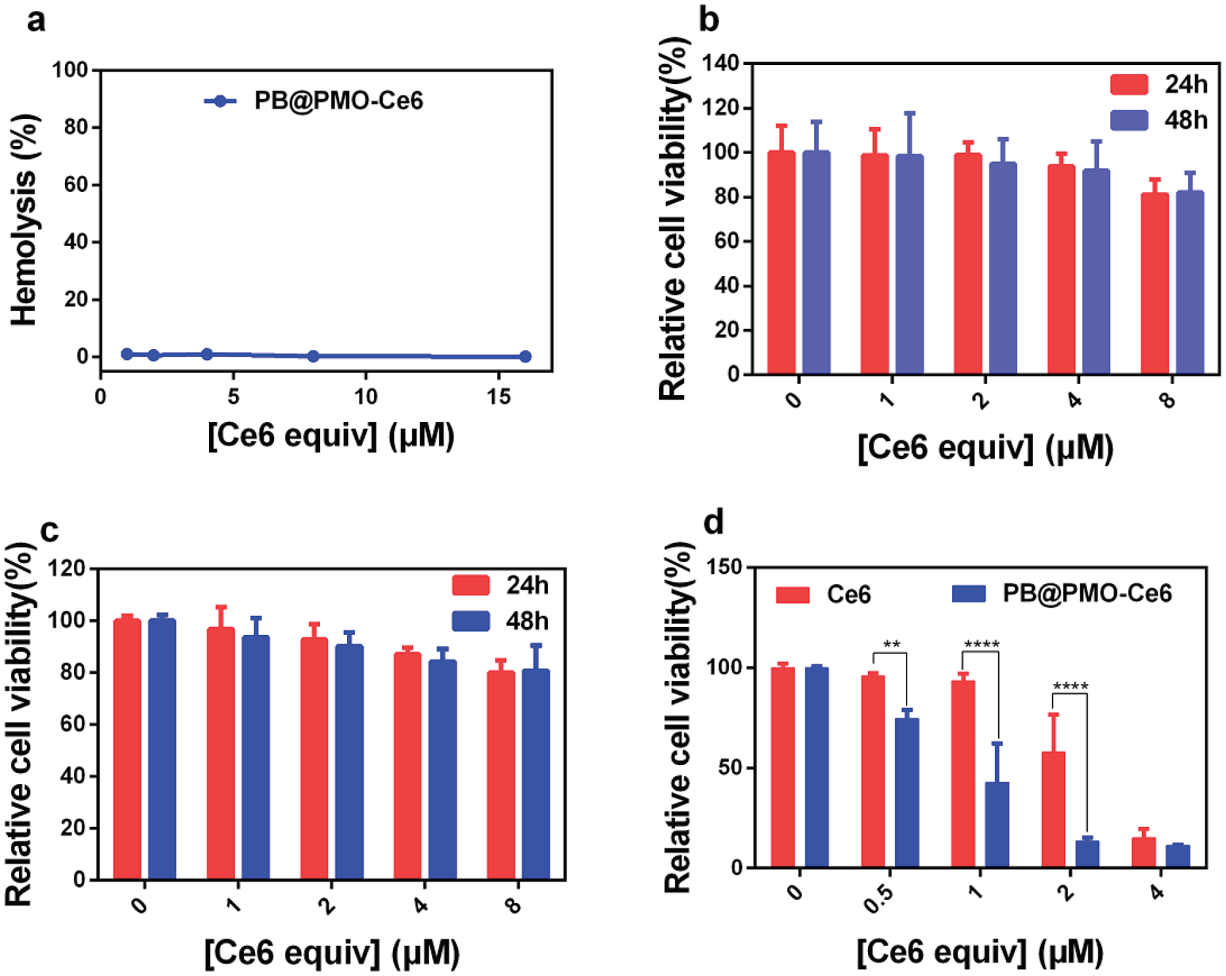
Cytotoxicity. a) The hemolytic activity of the PB@PMO‐Ce6 at concentrations of 1–16 × 10^−6^ m [Ce6 equiv.]. b) Relative viability of HUVEC incubated with PB@PMO‐Ce6 at different concentrations for 24 and 48 h. c) Relative viability of U87MG cells incubated with PB@PMO‐Ce6 at different concentrations for 24 and 48 h. d) Relative viabilities of U87MG cells incubated with Ce6 or PB@PMO‐Ce6 at different concentrations and then received a laser irradiation (660 nm, 1 W cm^−2^, 5 min).

Even at the nanoparticle concentration up to 16 × 10^−6^ m [Ce6 equiv.] the hemolytic activity of the PB@PMO‐Ce6 nanoparticles is as low as 0.15% (**Figure** **4**a). The relative viabilities of U87MG cells and human umbilic vein endothelial cells (HUVEC) all remain over 80% even after incubation with PB@PMO‐Ce6 at a concentration up to 8 × 10^−6^ m [Ce6 equiv.] (Figure 4b,c), indicating a good biocompatibility. Next, we tested the photodynamic effects on inducing cell death. After continual irradiation by a 660 nm laser for 5 min, the relative viabilities of U87MG cells decrease with the photosensitizer concentrations after the treatment with both Ce6 and PB@PMO‐Ce6 (Figure 4d). Notably, the relative cell viabilities are significantly reduced when treated with PB@PMO‐Ce6 than treated with Ce6 at the Ce6 concentrations of 0.5 × 10^−6^, 1 × 10^−6^, and 2 × 10^−6^ m (Figure 4d).

### 2.5 In Vivo MR/PA Imaging

**Figure 5:**
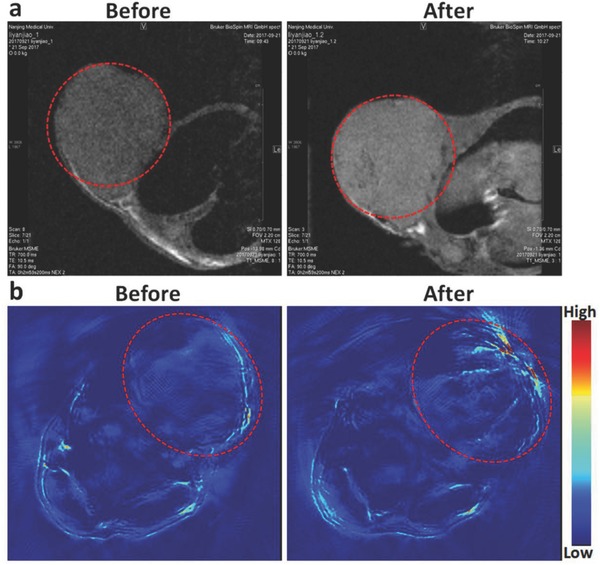
MR images and PA images of tumor‐bearing mouse. a) T1‐weighted MR images and b) PA images of tumor‐bearing mouse before and after the administration of PB@PMO‐Ce6 (80 × 10^−6^ m Ce6 equiv., 200 µL). The tumors are highlighted by circles.

Because the PB@PMO‐Ce6 is with a PB core, it is able to achieve MR and PA imaging. After injection of nanoparticles, it is observed that MR signals of tumor enhance obviously and homogeneously (**Figure** **5**a), which indicates the potential of PB@PMO‐Ce6 as a contrast material for MR imaging. The PA signal after injection also enhances mainly on the tumor surface area (Figure 5b). The unhomogeneous PA signal is most likely attributed to the limited penetration of laser.

### 2.6 PDT In Vivo

**Figure 6:**
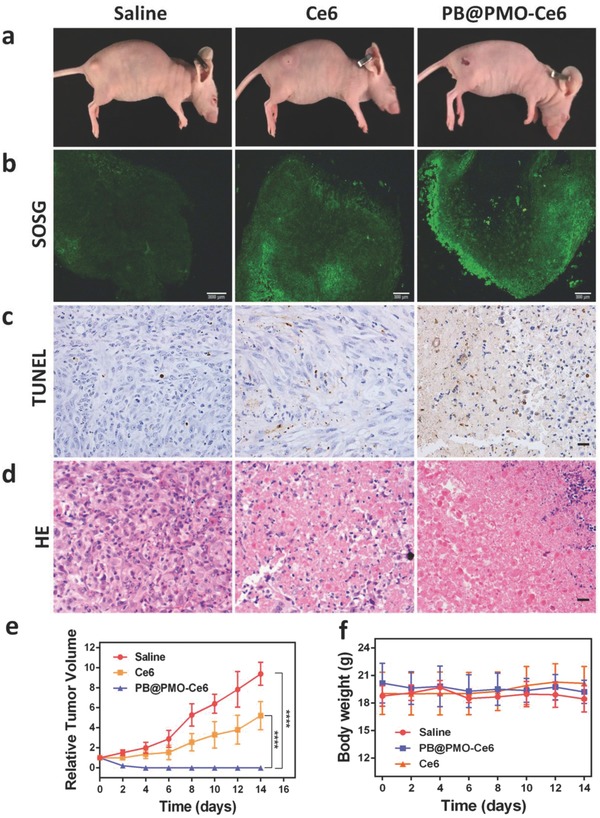
PDT in vivo. a) Representative photos of tumor‐bearing mice from different groups after treatment. b) SOSG staining in tumor sections for ^1^O_2_ detection (scale bar, 200 µm) after injection of SOSG‐contained saline, Ce6, or PB@PMO‐Ce6 into the tumors and laser exposure; c) TUNEL staining in tumor sections from different groups to evaluate the efficacy of PDT (scale bar, 20 µm); d) H&E staining in tumor sections from different groups to assess the PDT efficacy (scale bar, 20 µm); e) Relative tumor volume of tumor‐bearing mice treated with saline, Ce6, or PB@PMO‐Ce6 and laser irradiation. f) Body weight of the mice from different groups to determine the biocompatibility of PB@PMO‐Ce6 nanoparticles in vivo.

**Figure 7:**
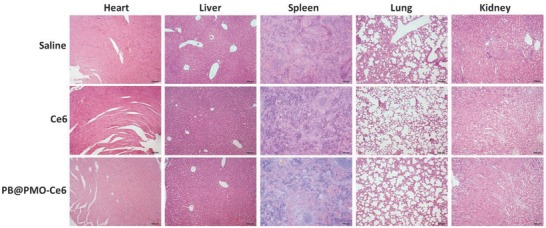
H&E images of major organs of tumor‐bearing mice from different groups (scale bar, 100 µm).

The efficacy of PDT in vivo was investigated by intratumoral injection of the nanoparticles into U87MG tumor‐bearing mice and laser irradiation. Apparent anticancer effect in PB@PMO‐Ce6 treated group is observed 2 d posttherapy with tumor necrosis, compared to the pale but unbroken tumor surface of Ce6 treated mouse and no obvious tumor change in saline group (**Figure** **6**a). The in vivo ^1^O_2_ generation was evaluated by observing frozen section after injection of mixtures of singlet oxygen sensor green (SOSG) with saline, Ce6 or PB@PMO‐Ce6, respectively. After laser irradiation, tumors treated with PB@PMO‐Ce6 show stronger green fluorescence than tumor treated with Ce6, while tumors treated with saline show weak fluorescence (Figure 6b), which indicates that higher singlet oxygen generation by PB@PMO‐Ce6 is realized in vivo. The PDT efficacy were further evaluated by analyzing tumor cell apoptosis and coagulative necrosis using TUNEL and H&E staining on tumor histological sections at day 2. More apoptotic cells in TUNEL staining sections from PB@PMO‐Ce6 group are found compared to Ce6 group (Figure 6c). It is noticed that necrosis occurred in most tumor cells of the mice treated with PB@PMO‐Ce6 and irradiation by a 660 nm laser (Figure 6d). In the group of Ce6, there is incomplete tumor cell necrosis, suggesting the enhanced efficacy in destroying tumor cells by PB@PMO‐Ce6 (Figure 6d). We further evaluated PDT effects by comparing relative tumor volumes of mice form different groups. The tumor volumes of mice in saline and Ce6 groups both increase along time (Figure 6e). The tumor growth shows a slower rate for Ce6 group, which indicates the limited tumor inhibition of free Ce6 for PDT. While in PB@PMO‐Ce6 group, the tumors are totally broken and form scabs 2 d posttherapy, which last until the end of experiment without obvious recurrence (Figure 6e). At day 14, the relative tumor volumes of mice receiving PB@PMO‐Ce6 treatment and 660 nm laser irradiation are significantly inhibited compared to groups of saline (0 vs 9.4 ± 1.2, *p* < 0.0001) and Ce6 (0 vs 5.2 ± 1.4, *p* < 0.0001) (Figure 6e). The improved efficacy of the PB@PMO‐Ce6 group is attributed to the self‐supplying O_2_ and efficiently generation of ROS, indicating the potential of the PB@PMOs‐Ce6 nanosystem for tumor photodynamic treatment. In order to assess the biocompatibility of PB@PMO‐Ce6 nanoplatform in vivo, the body weight and H&E staining on histological sections of major organs were analyzed. There is no significant reduction in the body weight of mice from all groups after treatment (Figure 6f). No obvious pathological changes are present on the main tissues after injection of the saline, Ce6 and PB@PMO‐Ce6 intravenously for 14 d (**Figure** **7**). These results indicate the safety and the potential clinic application of the PB@PMO‐Ce6 to enhance PDT efficacy.

## 3 Conclusion

In order to overcome hypoxia during PDT, we synthesized a safe, simple, integrated, and multifunctional nanoplatform, PB@PMO‐Ce6, to achieve the enhancement of PDT efficacy by first making use of the catalase‐like activity of PB. PB and PMO are both with excellent biocompatibility,^28^ which makes this nanoplatform suitable for application in vivo. The nanoparticle presents well‐defined core–shell structure, uniform diameter (105 ± 12 nm), and high biocompatibility. Owing to the PB cores and conjugated photosensitizer, the nanoparticles can effectively catalyze abundant but undesirable H_2_O_2_ into O_2_ and sequentially generate more ROS for PDT in the same system. A higher level of singlet oxygen and better therapeutic efficacy are present in group of PB@PMO‐Ce6 than Ce6 only both in vitro and in vivo, which indicates the ability of the nanosystem to enhance PDT. There are no obvious weight loss and organs damage by analyzing body weight and H&E staining sections of important organs of mice, which ensure the safety of this nanoparticles. Additionally, the preliminary PA and MR imaging results support its potentials to act as a contrast agent for imaging. The results from our study highlight the potential of the PB@PMO‐Ce6 nanoplatform to enhance PDT efficacy by elevating O_2_ and ROS in a highly integrated and simple nanosystem.

## 4 Experimental Section

*Chemicals and Reagents*: Potassium hexacyanoferrate (II) trihydrate (K_4_[Fe(CN)_6_] · 3H_2_O), iron (III) chloride anhydrous (FeCl_3_), tetraethyl orthosilicate (TEOS), hexadecyltrimethyl ammonium bromide (CTAB, 25wt%), dioxane, and triphenylphosphine were obtained from Sinopharm Chemical Reagent Co., Ltd. (Shanghai, China). Citric acid monohydrate, *N*,*N*‐dimethylformamide (DMF), anhydrous ethanol, concentrated ammonia aqueous solution (25 wt%), and hydrogen peroxide (30 wt%) were obtained from Nanjing Chemical Reagent Co., Ltd. (Nanjing, China). Acetone was acquired from Nanjing Aojia Chemical Co., Ltd. (Nanjing, China). TESPTS, *N*‐Carbamoylmaleimide, *N*‐hydroxysulfosuccinimide sodium salt (NHS), *N*‐(3‐dimethylaminopropyl)‐*N*′‐ethylcarbodiimide hydrochloride (EDC), Chlorin e6, and 2′,7′‐dichlorofluorescin diacetate (DCFH‐DA) were bought from Sigma‐Aldrich (St. Louis, MO, USA). Concentrated hydrochloric acid (HCl) (37%) was obtained from Shanghai Jiuyi Chemical Reagent Co., Ltd. (Shanghai, China). Tris(4,7‐diphenyl‐1,10‐phenanthroline)rutheniuM(II) dichloride (Ru(dpp)_3_Cl_2_) was obtained from Meryer (Shanghai) Chemical Technology Co., Ltd. (Shanghai, China). SOSG was purchased from Invitrogen (USA). Deionized water (Millipore) with a resistivity of 18 MΩ cm was used for all experiments. Dimethyl sulfoxide, Dulbecco's Modified Eagle's Medium (DMEM) and cell counting kit‐8 (CCK‐8) were bought from Nanjing Keygen Biotech. Co., Ltd. (Nanjing, China). Trypsin‐EDTA (0.25%), heat‐inactivated fetal bovine serum (FBS), and penicillin‐streptomycin solution were bought from Gibco Laboratories (NY, USA). U87MG cells were acquired from American Type Culture Collection (ATCC, Manassas, VA).

*Preparation*: The PB nanoparticles were first prepared using the method reported previously with a little change.^21^ Typically, K_4_[Fe(CN) _6_] aqueous solution (1.0 × 10^−3^ m, 40 mL) containing citric acid (0.9 mmol) was dropwisely added into FeCl_3_ aqueous solution (0.5 × 10^−3^ m, 40 mL) containing citric acid (0.9 mmol) under stirring (1200 rpm) at 60 °C to produce a clear, bright blue dispersion. The particles were collected by adding acetone (80 mL), centrifuging (13 000 rpm, 30 min), and washing thrice with the same volume of water and acetone. Then, a mixed solution containing PB nanoparticles (0.4 mg), CTAB (0.16 g), ethanol (25 mL), and water (80 mL) was stirred (980 rpm) at 35 °C for 1 h. A mixture of TEOS (100 µL) and TESPTS (10 µL) was then quickly added. The solution was stirred (980 rpm) at 35 °C for 48 h after adding concentrated ammonia aqueous solution (10 µL). Then the products were centrifugated and washed thrice using ethanol. The CTAB was extracted from the acquired products thrice in a mixed solution (60 mL) containing concentrated HCl (37%) and ethanol (volume ratio = 1: 500) at 60 °C for 3 h.^34^ After washing and drying, the PB@PMOs were acquired. The disulfide bonds contained in PMOs were reduced to thiol groups using the reported method.^21^ Briefly, a mixture of PB@PMOs (5 mg), triphenylphosphine (0.1 g), water (0.9 mL) and dioxane (3.3 mL) was heated to 40 °C under stirring (500 rpm) and added with concentrated HCl (20 µL) under nitrogen for two hours. The PB@PMOs with thiol groups were obtained and washed thrice using ethanol. To link amino, the above obtained products were added to DMF (1.5 mL) containing NH_2_‐maleimide (10 mg) and shaken for 24 h at room temperature. After centrifugation and washing, the amidogen groups contained PB@PMO was obtained. To modifying Ce6, the carboxyl groups contained Ce6 (0.5 mg mL^−1^, 0.4 mL) was first activated by EDC (1 mg mL^−1^, 0.24 mL) and NHS (1 mg mL^−1^, 0.28 mL) under shaking at room temperature for 2 h, and was added into amine groups contained PB@PMOs (500 µL). After reaction for 24 h, the Ce6 grafted PB@PMO (PB@PMO‐Ce6) was obtained by centrifugation and washing thrice using water.

*Characterization*: An HT7700 microscope (Hitachi, Japan) was used to capture the TEM images. The measurement of hydrodynamic sizes and zeta potential were performed by a Brookhaven analyzer (Brookhaven Instruments Co., Holtsville, USA). The UV–vis spectra of different materials were measured by a Lambda 35 UV–vis spectrophotometer (PerkinElmer, Inc., USA). The absorbance at 404 nm was used as a marker for the successful conjugation of Ce6. To estimate the amount of Ce6 conjugated on each PB@PMO, the PB@PMO‐Ce6 was dissolved in water and quantified by using Ce6 UV calibration curve at 404 nm. FT‐IR spectra were obtained using a Nicolet Nexus 870 spectrometer (Nicolet Instruments Inc. Madison, USA). Nitrogen sorption isotherms were acquired by a Micromeritics TriStar II 3020 analyzer (Micromeritics Instruments Corporation, USA). The specific surface areas and pore size were calculated by the Brunauer–Emmett–Teller and Barrett–Joiner–Halenda method, respectively.

*Evaluating Catalase‐Like Activity of PB@PMO‐Ce6*: In order to verify the generation of oxygen bubbles, phosphate buffered solution (PBS) (5 mL), Ce6 (1 × 10^−6^ m, 5 mL), and PB@PMO‐Ce6 (1 × 10^−6^ m Ce6 equiv., 5 mL) were respectively determined in three 15 mL centrifuge tubes with or without H_2_O_2_ (3.0 wt%, final concentration) at 37 °C for 30 min. The centrifuge tubes were then photographed.

*To Quantify the Generation of Oxygen*: PBS (100 µL), Ce6 (1 × 10^−6^ m, 100 µL) and PB@PMO‐Ce6 (1 × 10^−6^ m Ce6 equiv., 100 µL) were respectively added in 96‐well plate with 3.0 wt% H_2_O_2_ (final concentration) and [(Ru(dpp)_3_)]Cl_2_ (10 µg mL^−1^, 10 µL) and incubated at 37 °C for 30 min. [(Ru(dpp)_3_)]Cl_2_ is a commercial oxygen sensing probe, the fluorescence of which is strongly reduced by oxygen molecular.^25^, ^35^ Then, the emission at 620 nm was recorded for each well after excitation at 488 nm by a microplate reader (Infinite M200 pro, Tecan, Switzerland).

*For Determining the Generation of Oxygen at Different Concentrations of H_2_O_2_*: PB@PMO‐Ce6 (1 × 10^−6^ m Ce6 equiv., 100 µL) were respectively dispersed in 96‐well plate with 0, 1.5, 3, and 6 wt% H_2_O_2_ (final concentration) and [(Ru(dpp)_3_)]Cl_2_ (10 µg mL^−1^, 10 µL) and incubated at 37 °C for 30 min. The emission at 620 nm was recorded for each well after excitation at 488 nm.

*To Detect the Generation of Oxygen in Cell*: U87MG cells were planted into a 96‐well plate (1 × 10^5^ cells per well) in DMEM (0.2 mL) containing 10% FBS at 37 °C and 5% CO_2_ for 12 h. The [(Ru(dpp)_3_)]Cl_2_ (10 µg mL^−1^, 20 µL) was added. Twelve hours later, the cells were washed once using PBS and DMEM (200 µL) was added. Then, PBS, Ce6 or PB@PMO‐Ce6 was respectively added to the wells. The final concentration of Ce6 was 1 × 10^−6^ m. The cells were further incubated for 24 h. After washed once by PBS and added with DMEM (100 µL), the cells were excited at 488 nm and the emission at 620 nm was recorded.

*Detection of Reactive Oxygen Species In Vitro*: Singlet oxygen was measured with widely used highly specific probe SOSG.^36^ Upon the presence of ^1^O_2_, the intrinsic fluorescence of SOSG is restored, resulting in increased fluorescence.^36^ First, PB@PMO‐Ce6 (100 µL) and SOSG (50 × 10^−6^ m, 10 µL) were mixed in 1.5 mL centrifuge tubes with or without 3 wt% H_2_O_2_ (final concentration). The final concentration of Ce6 was 1 × 10^−6^ m. After irradiation (660 nm, 1 W cm^−2^) for different time periods (0, 1, 3, 5, 10 min), the fluorescence intensity was detected using a microplate reader (Infinite M200 pro, Tecan, Switzerland) (λ_ex_ = 490 nm, λ_em_ = 530 nm). The experiments for each group were run in triplicate.

*To Determine the Generation of Reactive Oxygen Species in Cells*: U87MG cells (1 × 10^5^ cells per well) were seeded into a 96‐well plate in DMEM (0.2 mL) containing 10% FBS at 37 °C and 5% CO_2_ for 12 h. Then, PBS, Ce6 or PB@PMO‐Ce6 was added to the wells. The final concentration of Ce6 was 1 × 10^−6^ m. The cells were further incubated for 12 h. After washed once by PBS, the cells were added with DMEM (100 µL) containing DCFH‐DA (20 × 10^−6^ m) and were further incubated for 4 h. Then, the cells were washed once using PBS and irradiated by a laser (660 nm, 1 W cm^−2^) for 5 min per well. Subsequently, the cells were excited at 485 nm and the emission was recorded at 525 nm by a microplate reader (Infinite M200 pro, Tecan, Switzerland).

*The Flow Cytometry Analysis Was Also Performed to Determine the Generation of ROS*: U87MG cells (4 × 10^5^ cells per well) were seeded into a 12‐well plate for 12 h and then incubated respectively with PBS, Ce6 or PB@PMO‐Ce6 with a 1 × 10^−6^ m final concentration of Ce6. Twelve hours later, the cells were washed once by PBS and incubated in DMEM (1 mL) containing DCFH‐DA (20 × 10^−6^ m) for additional 4 h. Then, the cells were washed once by PBS and irradiated using a laser (660 nm, 1 W cm^−2^) for 5 min per well. The cells were washed and suspended in PBS (500 µL) and analyzed using a flow cytometry (Beckman coulter, NJ, USA). Data were obtained and analyzed using the FLOWJO program.

*Biocompatibility Studies*: The cytotoxicity of the PB@PMO‐Ce6 was evaluated using U87MG cells and normal cells, HUVEC, respectively. After seeded into a 96‐well plate (1 × 10^5^ cells per well) for 12 h, the cells were incubated with the PB@PMO‐Ce6 at different concentrations (0–8 × 10^−6^ m Ce6 equiv.) for 24 h or 48 h at 37 °C. Five wells were performed in parallel for each concentration. After that, the CCK8 (20 µL) was added and incubated for an additional 4 h. Finally, the absorbance was recorded at 450 nm using a microplate reader (Infinite M200 pro, Tecan, Switzerland). The cell viability was calculated using the reported method.^37^

*The Hemocompatibility of PB@PMO‐Ce6 Was Evaluated Using the Previously Reported Method*:^38^ Human blood containing heparin was acquired according to an informed content of the local Medical Research Ethics Committee. A mixture of normal saline (NS, 1 mL) and whole blood (1 mL) was centrifuged (2000 rpm, 5min) to get the separated red blood cells. The red blood cells were washed until the supernatant turn to colorless, and then dispersed in NS (5 mL). Then, RBC suspension (200 µL) was added to PB@PMO‐Ce6 (800 µL) with different concentrations (1–16 × 10^−6^ m Ce6 equiv.) in NS. As the negative or positive control, RBC suspension (200 µL) was respectively added in NS (800 µL) or deionized water (800 µL). The mixture was gently shook and then maintained at 37 °C for 2 h. After centrifugation, the absorbance value of supernatant at 570 nm was recorded and the absorbance at 630 nm was used as reference. The hemolysis was calculated according to previously reported work.^38^

*In Vitro PDT*: U87MG cells (1 × 10^5^ cells per well) were planted into a 96‐well plate in DMEM (200 µL) for 12 h. Then, the cells were incubated with the Ce6 or PB@PMO‐Ce6 at different concentrations (0–4 × 10^−6^ m Ce6 equiv.) for 24 h at 37 °C. Five wells were performed in parallel for each concentration. After washed once with PBS, the cells were added with fresh DMEM (100 µL) and irradiated by a laser (660 nm, 1 W cm^−2^) for 5 min per well. The CCK8 (10 µL) was then added and incubated for another 4 h. Finally, the absorbance was recorded at 450 nm and the relative cell viability was calculated referring to previously reported method.^21^

*Animal Model Establishment*: All animal experiments have obtained the permission from the local institutional ethical committee. The Balb/c nude mice (female, 5–7 week old) were bought from Nanjing Peng Sheng Biological Technology Co. Ltd. U87MG cells (5 × 10^6^, 100 µL) were subcutaneously planted into the mice at their lower flanks or right lower limbs to establish xenograft models. The tumors located at the lower flanks of mice were used for MR and PA imaging because those lesions usually grew faster which facilitated images observation. The right lower limb tumors were selected for evaluating PDT in vivo.

*MR and PA Imaging In Vivo*: After the model of tumor‐bearing mice was established, PB@PMO‐Ce6 (80 × 10^−6^ m Ce6 equiv., 200 µL) was administrated by intratumoral injection for the mice. MR imaging was collected by using the Biospec 7T/20 USRMRI instrument (Bruker BioSpin, Germany). PA images were captured on a multispectral photoacoustic tomography imaging system (iThera Medical inVision 256‐TF, Germany)

*PDT In Vivo*: When the maximum diameters of tumor became about 5–8 mm, a mixture of SOSG (50 × 10^−6^ m, 25 µL), saline (25 µL), Ce6 (25 µL) or PB@PMO‐Ce6 (25 µL) was directly injected into tumors. The final concentration of Ce6 was 80 × 10^−6^ m. Subsequently, a laser irradiation (660 nm, 1 W cm^−2^) was performed on the saline, Ce6 and PB@PMO‐Ce6 groups for 2 min. Then, tumors from each group were harvested and cryosectioned onto slides. The fluorescence emission of the SOSG‐staining sections was recorded by using a fluorescence microscope (Olympus IX71, Nanjing, China).

Furthermore, the tumor‐bearing mice with the tumor maximum diameter of about 5–8 mm were randomly divided into three groups (*n* = 6 per group), as follows: group 1: saline alone; group 2: Ce6; group 3: PB@PMO‐Ce6. First, saline (50 µL), Ce6 (50 µL) and PB@PMO‐Ce6 (50 µL) was injected into tumor of each mouse respectively from group 1 to 3. The final concentration of Ce6 was 80 × 10^−6^ m. Thirty minutes later, the tumor region of each mouse received laser irradiation (660 nm, 1 W cm^−2^) for 2 min. At day 2, tumors from each group were collected and performed with TUNEL and H&E staining following the manufacturer's instructions. Tumor sizes and mice body weight were measured and recorded every 2 d from day 0 to 14 for those remaining mice from the three groups (*n* = 5 per group). The tumor size was recorded as the maximum width (*X*) and length (*Y*), and the tumor volume (*V*) was calculated using the following equation: *V* = (*X* ^2^ *Y*)/2. The relative tumor volume was used to reflect changes of tumor volume for each mouse and was calculated by normalizing the tumor volume at day *X* to their initial tumor volume. In order to evaluate the biocompatibility in vivo, saline (100 µL), Ce6 (100 µL), and PB@PMO‐Ce6 (100 µL) were respectively injected intravenously into additional mice from the three groups (*n* = 2 per group). The final concentration of Ce6 was 80 × 10^−6^ m. After 14 d, the heart, liver, lung, kidney, and spleen of mice from different group were collected for histological analysis.

*Statistical Analysis*: Experimental results were shown as the mean ± standard deviation. Statistical analysis was performed by using GraphPad Prism 6 (GraphPad Software Inc., CA, USA). The difference of the generation of oxygen and singlet oxygen, cellular viability and tumor volume of different groups was analyzed by the analysis of variance test followed by the post hoc TukeyHSD test. *p* < 0.05 was considered to indicate a statistical difference. Probabilities as *p* < 0.05 (*), *p* < 0.01 (**), *p* < 0.001 (***), *p* < 0.0001 (****), and no significance (n.s.) were labeled in figures.

## Conflict of Interest

The authors declare no conflict of interest.

## Supporting information

Supplementary
