# Supplementary material for: Oxygen‐Evolving Mesoporous Organosilica Coated Prussian Blue Nanoplatform for Highly Efficient Photodynamic Therapy of Tumors
Source: Adv Sci (Weinh). 2018 Feb 22;5(5):1700847. doi: 10.1002/advs.201700847 (PMC5980201; doi:10.1002/advs.201700847)
Supplement: Supplementary file 1 — Supplementary [file ADVS-5-1700847-s001.pdf]

Copyright WILEY-VCH Verlag GmbH & Co. KGaA, 69469 Weinheim, Germany, 2018.

Supporting Information

for *Adv. Sci.*, DOI: 10.1002/advs.201700847

Oxygen-Evolving Mesoporous Organosilica Coated Prussian Blue Nanoplatform for Highly Efficient Photodynamic Therapy of Tumors

Zhen Lu Yang, Wei Tian, Qing Wang, Ying Zhao, Yun Lei Zhang, Ying Tian, Yu Xia Tang, Shou Ju Wang, Ying Liu, Qian Qian Ni, Guang Ming Lu, Zhao Gang Teng,* and Long Jiang Zhang**

Supporting Information

Oxygen-evolving Mesoporous Organosilica Coated Prussian Blue Nanoplatform for Highly Efficient Photodynamic Therapy of Tumor

Zhen Lu Yang, Wei Tian, Qing Wang, Ying Zhao, Yun Lei Zhang, Ying Tian, Yu Xia Tang, Shou Ju Wang, Ying Liu, Qian Qian Ni, Guang Ming Lu, Zhao Gang Teng*, and Long Jiang Zhang**

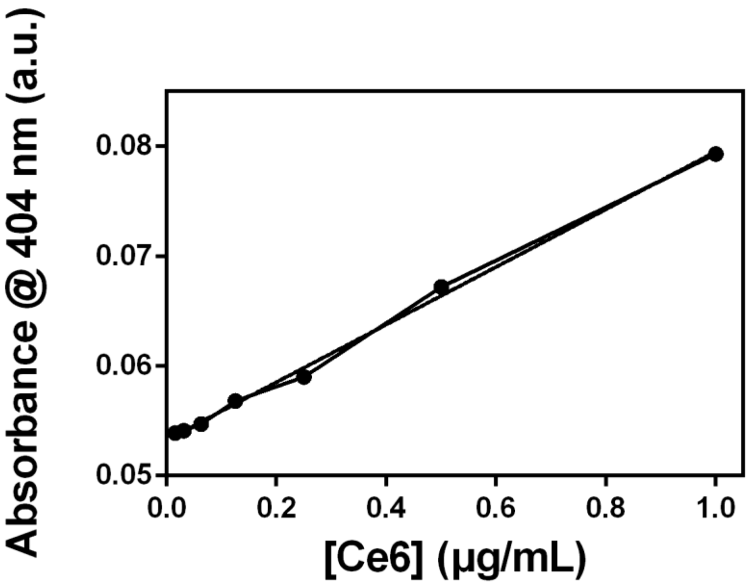

Figure S1: **The linear relationship between concentration and absorbance of Ce6 at 404 nm.**

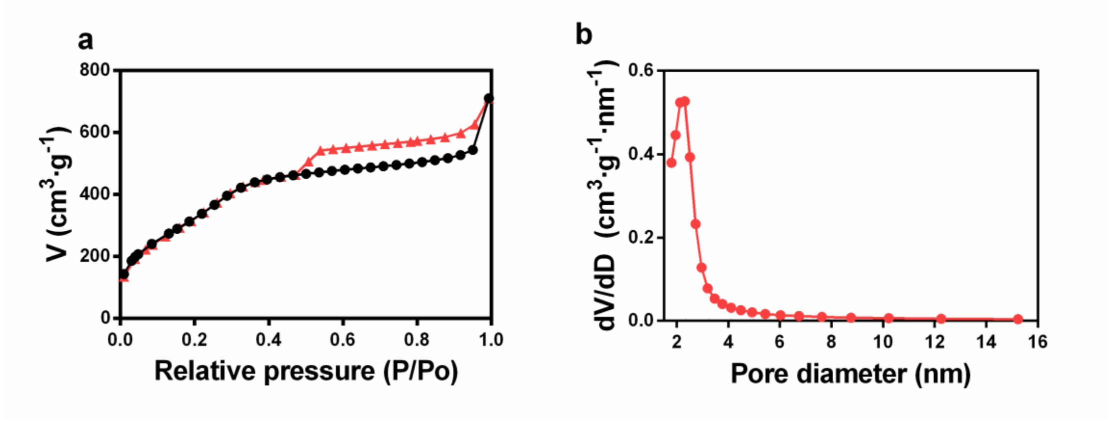

Figure S2: (a) Nitrogen sorption isotherms of PB@PMO. (b)Pore size distribution curve.
